# Field Validation of a Transcriptional Assay for the Prediction of Age of Uncaged *Aedes aegypti* Mosquitoes in Northern Australia

**DOI:** 10.1371/journal.pntd.0000608

**Published:** 2010-02-23

**Authors:** Leon E. Hugo, Peter E. Cook, Petrina H. Johnson, Luke P. Rapley, Brian H. Kay, Peter A. Ryan, Scott A. Ritchie, Scott L. O'Neill

**Affiliations:** 1 School of Biological Sciences, University of Queensland, Brisbane, Australia; 2 Queensland Institute of Medical Research and Australian Centre for International and Tropical Health and Nutrition, Herston, Australia; 3 School of Public Health, Tropical Medicine and Rehabilitation Sciences, James Cook University, Cairns, Australia; 4 Tropical Population Health Services, Queensland Health, Cairns, Australia; Duke University-National University of Singapore, Singapore

## Abstract

**Background:**

New strategies to eliminate dengue have been proposed that specifically target older *Aedes aegypti* mosquitoes, the proportion of the vector population that is potentially capable of transmitting dengue viruses. Evaluation of these strategies will require accurate and high-throughput methods of predicting mosquito age. We previously developed an age prediction assay for individual *Ae. aegypti* females based on the transcriptional profiles of a selection of age responsive genes. Here we conducted field testing of the method on *Ae. aegypti* that were entirely uncaged and free to engage in natural behavior.

**Methodology/Principal Findings:**

We produced “free-range” test specimens by releasing 8007 adult *Ae. aegypti* inside and around an isolated homestead in north Queensland, Australia, and recapturing females at two day intervals. We applied a TaqMan probe-based assay design that enabled high-throughput quantitative RT-PCR of four transcripts from three age-responsive genes and a reference gene. An age prediction model was calibrated on mosquitoes maintained in small sentinel cages, in which 68.8% of the variance in gene transcription measures was explained by age. The model was then used to predict the ages of the free-range females. The relationship between the predicted and actual ages achieved an *R^2^* value of 0.62 for predictions of females up to 29 days old. Transcriptional profiles and age predictions were not affected by physiological variation associated with the blood feeding/egg development cycle and we show that the age grading method could be applied to differentiate between two populations of mosquitoes having a two-fold difference in mean life expectancy.

**Conclusions/Significance:**

The transcriptional profiles of age responsive genes facilitated age estimates of near-wild *Ae. aegypti* females. Our age prediction assay for *Ae. aegypti* provides a useful tool for the evaluation of mosquito control interventions against dengue where mosquito survivorship or lifespan reduction are crucial to their success. The approximate cost of the method was US$7.50 per mosquito and 60 mosquitoes could be processed in 3 days. The assay is based on conserved genes and modified versions are likely to support similar investigations of several important mosquito and other disease vectors.

## Author Summary

Once infected with dengue virus, a female *Aedes aegypti* mosquito must survive longer than twelve days before it can transmit the virus to an uninfected person. New dengue control strategies therefore aim to circumvent dengue transmission using entomopathogenic microorganisms that shorten mosquito lifespan. Accurate methods to determine the age of individual mosquitoes are required for these and other mosquito control interventions. We have previously shown that mosquito age can be predicted from the transcription of specific genes. Here we demonstrate that this can be achieved under natural conditions when mosquitoes are uncaged and free to engage in natural behavior. To do this, we produced “free-range” female mosquitoes by releasing 8007 mosquitoes at an isolated location and recapturing the females of known ages. We developed an age prediction model from gene transcription measures of mosquitoes maintained in small “sentinel cages” maintained onsite. We then used this model to predict the ages of the free-range mosquitoes, based on their own transcription measures. Age predictions were robust to physiological changes associated with blood feeding and egg development. We show that the technique could be applied to identify a 50% reduction in mosquito population survival that is expected from infection with entomopathogenic *Wolbachia* bacteria.

## Introduction

The survival of mosquitoes to a relatively old age is required for the transmission of mosquito-borne diseases. Mosquito-borne pathogens such as dengue viruses and malaria parasites require a period of development or multiplication inside the mosquito (extrinsic incubation period; EIP) before transmission can occur. Female mosquitoes ingest the pathogen when taking a blood meal from an infected host. The pathogen must then penetrate the midgut, escape from the midgut, multiply and disseminate through the mosquito before infecting the salivary glands. Transmission may then occur when the female subsequently bites a naïve host. For many of the world's most important mosquito-borne diseases (malaria, dengue and lymphatic filariasis), the EIP of the parasite or virus is long relative to the lifespan of the mosquito vector. The EIP of the dengue viruses in the primary mosquito vector, *Aedes aegypti*, is approximately 12–16 d [1]. Landmark epidemiological studies identified mosquito survival as a target for the prevention of mosquito borne disease and a sensitive indicator of disease activity [2]–[4]. However, the importance of mosquito longevity has seldom been directly tested because few tools exist that can accurately determine the age of wild caught mosquitoes.

Mosquito control strategies adopting various microbial agents aim to reduce mosquito longevity to impact disease transmission [5]–[9]. Successful infection of *Ae. aegypti* with a life-shortening strain of the intracellular bacteria *Wolbachia* has recently been reported [9]. The infection causes a 50% reduction in *Ae. aegypti* longevity, is maternally inherited and has the capacity to be driven through wild mosquito populations through the mechanism of cytoplasmic incompatibility. Implementation of these strategies will require rapid and high throughput age determination of the targeted mosquito vectors to evaluate the efficacy of control. Traditional dissection based methods of age grading mosquitoes fall well short of the required accuracy and throughput required, and biochemical approaches such as the measurement of cuticular hydrocarbons lose accuracy beyond 15 d old [10]–[13].

We previously reported a method of predicting the age of adult female *Ae. aegypti* mosquitoes using the transcriptional profiles of age responsive genes [14],[15]. Quantitative Reverse Transcriptase PCR (qRT-PCR) was used to measure the transcriptional profiles of these genes from the head and thorax of individual mosquitoes. Age predictions were then derived from the transcriptional profiles using multivariate calibration. An initial validation of the method was performed on mosquitoes that had been maintained inside 13 m^3^ capacity dome tents positioned inside and adjoining an elevated residence in Cairns, northern Australia. An age prediction model incorporating eight genes was reported that facilitated age predictions of female *Ae. aegypti* to within ±5 d of their actual age up to 19 d of age. For large scale studies, a reduced model incorporating three genes was recommended. 74.99% of the variation in the transcription of these genes was explained by mosquito age. However, the activity of mosquitoes in cages may have been reduced, affecting their capacity to seek out preferred micro-habitats with unknown consequences for the aging assay. Similarly, the influence of major physiological changes during the mosquito blood feeding - egg development cycle is unknown. Physiological processes associated with the mosquito gonotrophic cycle involve extensive changes in gene expression [16]–[19].

Here we report the evaluation of transcriptional age grading on *Ae. aegypti* that were entirely uncaged as adult mosquitoes (called free-range mosquitoes). By conducting the experiment at an isolated homestead without resident *Ae. aegypti*, a large cohort of mosquitoes could be released unmarked and recaptured at known ages to 29 d old. To facilitate high throughput age grading, a multiplexed assay incorporating Taqman probes [20] was used for quantitative PCR analysis. Several physiological parameters (including body size, blood digestion and ovary development) were investigated as possible sources of variance in our age predictions. We have demonstrated that mosquito age estimates generated from transcriptional profiles under natural conditions can be applied to identify changes in mean mosquito population life expectancy. By incorporating Taqman probes, the method could be scaled-up to facilitate the expected increases in requirements for mosquito age grading when new mosquito control strategies are implemented.

## Materials and Methods

### Ethics statement

Human ethics approval for allowing colonized (dengue-free) mosquitoes to feed on the investigators was obtained from James Cook University (Human ethics approval H2250). Blood feeding was considered to cause a medium risk of allergic reaction and provision was in place that individuals were excluded if they reacted strongly to bites. Written consent was obtained acknowledging the right to refuse or withdraw.

### Mosquitoes

*Aedes aegypti* were collected as eggs from ovitraps set at Machans Beach (16° 51′ 14″ S, 145° 44′ 55″ E), a suburb of Cairns, Queensland, Australia. G1 eggs were hatched in hay-infused water and reared on a diet of dry adult cat food (Friskies; North Ryde, Australia) under ambient conditions and low densities to ensure synchronous development. Pupae were transported to a homestead in an isolated rainforest located 13 km from Cairns. The homestead consisted of a cluster of three buildings; a single story, one bedroom, unscreened wooden house and two open-sided shelters. *Ae. aegypti* were known to be absent from the site and this was confirmed by attempts to trap *Ae. aegypti* using three adult mosquito traps (BG-Sentinel traps; Biogents, Regensburg, Germany) at the site over a four week period before release. The pupae were randomly divided into a “free-range” group for release and a “sentinel-cage” group. A third group was used to determine the sex ratio (*n* = 83). The free-range pupae were transferred to open 9 L release containers that were maintained at the site for 24 hr. Over this time the adults emerged and dispersed. The number of adult *Ae. aegypti* released was estimated by counting the pupal exuviae in the release containers at the end of the release period. Mosquitoes were allowed free movement around the property and volunteers residing at the site provided blood meals at 2 d intervals. The property was supplemented with additional larval habitats (tyres, buckets and pot-plant bases) and all larval habitats/potential oviposition sites were flushed-out and cleaned every 5 to 6 d to prevent emergence of any adult mosquitoes. Fifteen resting adult females were recaptured at 2 d intervals from 1 to 29 d from various sites around the field house using mechanical aspirators. Temperature and humidity was recorded throughout the experiment using Hobo data loggers (Onset Computer Corporation, Pocasset, U.S.A). The sentinel cage group was placed into two cages (450×450×450 mm) that were maintained on-site for the duration of the experiment and 10 mosquitoes were sampled from these at 4 d intervals from 1 to 29 d. At the time of capture, mosquitoes were briefly anaesthetized at −20°C for 5 min. The heads and thoraces of individual mosquitoes were dissected from abdomens, wings and legs on a glass slide using fine tweezers and a scalpel. Heads and thoraces were rapidly placed in 300 µl of RNAlater (Ambion, Austin, U.S.A) and stored as per the manufacturer's protocol. Abdomens of recaptured mosquitoes were stored at −20°C until dissections were performed to determine physiological status. At the conclusion of sampling, mosquitoes were exhaustively removed by aspirator collections and using four BG-Sentinel traps and 18 sticky-ovitraps positioned around the homestead over a three week period. No *Ae. aegypti* were collected after the second week or have been observed since, indicating that the mosquitoes did not become established.

Various physiological characteristics of the free-range females were determined by dissection. Mating status was determined from the presence or absence of sperm in the spermathecae. The midgut was observed and females were classified as to whether blood was present or absent. Ovarian development was graded according to Christophers' stages [21] and parity by the presence or absence of ovary tracheolar skeins [22]. Wing length was used as a proxy for body size and was measured as the distance from the axial notch to the wing tip, excluding the fringe scales [23].

### Total RNA isolation

Samples were removed from RNAlater and transferred to 1.5 ml screw-capped plastic vials with a single 3 mm silica glass bead and 0.5 ml Trizol reagent (Invitrogen, Carlsbad, U.S.A.). Samples were mechanically homogenized in a Minibeadbeater (Biospec) for 1.5 min, transferred to 1.5 ml microfuge tubes and centrifuged at 17,000×g for 10 min at 4°C to pellet the chitinous material. The supernatant was transferred to a new 1.5 ml microfuge tube. Trizol extraction was performed according to manufacturer's instructions; however, isopropanol precipitation was performed overnight at −30°C. Total RNA pellets were reconstituted in 20 µl RNAse-free water. Total RNA was quantified by absorbance readings using a Nanodrop spectrophotometer (Biolab, Scoresby, Australia). Total RNA was standardized at 500 ng and treated with 0.2 U recombinant RNAse-free DNase (Roche) as per the manufacturer's protocol.

### Development of the multiplex qRT-PCR assay

A previous report [14] proposed a reduced set of three gene expression (GE) measures (*Aedes aegypti calcium binding protein* [*Ae-15848*; XM_001653412], *Aedes aegypti pupal cuticle protein 78E* [*Ae-8505*; XM_001656550] and *Aedes aegypti cell division cycle 20* [*cdc20; fizzy*][*Ae-4274*; XM_001664201]) all normalized to a housekeeping gene *Aedes aegypti 40S ribosomal protein S17* [*Ae-RpS17*; AY927787]). Primer and dual-labelled Taqman probe sets (Table S1) were designed for this gene set, using web-based assay design software *RealTimeDesign* (http://www.biosearchtech.com/products/probe_design.asp; Biosearch Technologies Novato, CA), to allow multiplex qRT-PCR assays to be developed. Gene-specific Taqman probes were labeled with different fluorophores that had minimal spectral overlap to minimize “cross-talk” between color channels. Four different fluorophores were used to label Taqman probes specific to each gene. Gene-specific labeling was incorporated into the Taqman assay to allow for the possibility of a triplex, excluding the housekeeping gene, or quadraplex assay to be designed. However, these initial attempts to co-amplify three or four PCR products were unsuccessful as discussed below. Instead, two duplex assays were optimized to co-amplify: (1) *Ae-RpS17* and *Ae-15848*, and (2) *Ae-4274* and *Ae-8505*. Multiplex reactions were validated by comparing the Ct values obtained from the duplex and single-plex assays across a 10^7^-fold dilution series (10^0^–10^7^ copies). The dilution series was constructed using a mixture of linearized plasmids containing inserts for each gene of interest. For the construction of plasmids, PCR products were amplified from a pool of *Ae. aegypti* cDNA, gel purified, ligated into pGEM-Teasy (Promega, Madison, U.S.A) and transformants cultured. Mini-preps were digested with *AatII* for 2 h to linearize plasmids, which were then quantified and serially diluted. All qRT-PCR assays contained 500 nM of each primer, 200 nM Taqman probe, 6 mM MgCl_2_ and 2 µl template cDNA, and were amplified with the following cycling conditions: 50°C, 2 min; 95°C, 2 min; then 50 cycles of 95°C for 10 s; 60°C for 20 s; fluorescence acquisition.

All qRT-PCR assays were run in triplicate on the Corbett Rotorgene 6000 real-time PCR platform (Corbett Research, Sydney, Australia). Ct values were calculated as the second derivative maximum of the fluorescence curve using the comparative quantification analysis module in the Rotorgene software (Corbett Research, version 1.7). Mean Ct values were calculated from replicate reactions and used to construct standard curves for single and duplex reactions. Single and duplex standard curves were analyzed with the ANCOVA procedure in SAS (version 9, SAS Institute, Cary, U.S.A.) to determine that they were comparable in terms of slope.

### Determining transcript abundance using qRT-PCR

Reverse transcription was performed using 500 ng of DNAse-treated total RNA, anchored oligo(DT)_20_ priming, 20 U RNaseOut (Invitrogen) and 100 U Superscript III reverse transcriptase (Invitrogen) based on the manufacturer's protocols. cDNA was diluted 5-fold to minimize the influence of PCR inhibitors. A random sample of 15 RT reactions were re-synthesized as negative RT controls (no reverse transcriptase). These were screened for genomic DNA contamination by standard PCR with *Ae-RpS17* primers (95°C, 3 min; 95°C, 30s; 60°C, 30s; 72°C, 1 min; 35 cycles; 72°C, 10 min). All RT negative controls tested negative. Transcriptional profiling of *Ae-RpS17*, *Ae-15848*, *Ae-8505* and *Ae-4274* was performed using the multiplex qPCR assay described above.

### Statistical analysis

ANOVA was applied to investigate the effects of adult mosquito age and confinement (sentinel cage versus free-range) on total RNA yield (µg) from the head and thorax of all *Ae. aegypti* females. Age was log_10_ transformed to account for curvature in the change in total RNA yield at younger ages. The influence of mosquito physiological parameters on total RNA yield was evaluated for free-range females. ANOVA was performed on total RNA yield with presence of blood in the midgut (no blood or some blood) and Christophers' ovarian development stage (stage I to V; stage G females were omitted due to the disproportionate statistical influence of this group) as factors and log-age and wing length as covariates. Estimated means for Christophers' ovarian stage groups were then calculated and compared. ANOVA was implemented in SPSS (SPSS Inc., Chigago, U.S.A).

Gene transcription measures for *Ae-15848*, *Ae-8505* and *Ae-4274* were normalized to the expression of the housekeeping gene (*Ae-RpS17*) by calculating log contrast values for each gene [15] (log_10_ of the ratio of the Ct value to the Ct value of *Ae-RpS17*). The effects of adult mosquito age, grouping, wing length, blood presence and ovary development on the log contrast gene transcription measures was determined using ANOVA as described above for total RNA yield.

An age prediction model was constructed using a multivariate analysis procedure that extracts a linear variable from multiple gene transcription measures [14],[15]. Briefly, log contrast values of test mosquitoes (sentinel cage females) were entered into canonical redundancy analysis to reduce the dimensionality of the data by creating new variables called redundancy variates. The age prediction calibration model was created by regression of the first redundancy variate against adult mosquito age for the sentinel cage females.

A non-parametric bootstrapping procedure was used to predict the age of each free-range female. This analysis was implemented in SAS (version 9.1; SAS Institute, Cary, U.S.A) using a SAS editor syntax that is provided in Cook et al. [15]. The sentinel cage dataset was input as the training dataset (*n* = 72) and log contrast values for all free-range females were input as the test dataset (*n* = 145). The mean of 1000 bootstrap age predictions for each free-range female was reported as its predicted age. Alternative models were investigated for the prediction of mosquito age from the transcriptional measures. A Poisson regression model with a logarithmic link function was applied because predictions were constrained to positive values. Mosquito age was analyzed so that the regression coefficients described the log of the relative risk of the independent variables (log contrast values for *Ae-15848*, *Ae-8505* and *Ae-4274* with or without total RNA yield). Poisson regression with logarithmic link models were implemented in WinBUGS [24]. In addition, the redundancy variate model was repeated as described but with total RNA yield as an additional independent variable. Sources of variance in age prediction accuracy of the redundancy variate three gene model were investigated by performing ANOVA on the age prediction residuals (predicted age minus actual age) with blood digestion and ovary development as factors and log-age and wing length as covariates.

The accuracy of mean life expectancy (*e_x_*) estimates that would be derived by applying our grading technique was investigated using Monte Carlo simulations. In particular, we tested our age grading method for the ability to differentiate two mosquito populations with a two-fold difference in mean life expectancy. This difference was chosen to test the ability to detect a 50% lifespan reduction induced by *Wolbachia* wMelpop infection. Two populations of 10,000 mosquitoes were created for which survival was described by exponential mortality models with *e_x_* set at 5 (probability of daily mortality [α] of 0.1) and 10 days (α = 0.2), respectively. Samples of 100, 200, 300, 400 and 500 mosquitoes of defined ages were randomly removed from the population. The age of each mosquito was predicted by randomly sampling from normal cumulative distributions of ages described by the mean and standard deviation of the experimentally predicted age estimates at each age. The distributions for even-aged mosquitoes were defined by the interpolated mean and standard deviations from the adjacent experimentally determined values. Mortality rates were estimated by calculating the regression coefficient of the natural log of the proportion of the population predicted to be within 24 hr age intervals against the age of each class. Age classes containing <3 mosquitoes were omitted from the calculations. Estimated *e_x_* values were then calculated (1/- α_estimated_). Sampling, age predictions and *e_x_* predictions were iterated 999 times using the Monte Carlo simulation function in the PopTools add-in in Microsoft Excel (http://www.cse.csiro.au/poptools/). Ninety five percent confidence intervals for *e_x_* were determined from the 2.5 and 97.5 percentiles of the resulting distributions.

## Results

Approximately eight thousand newly emerged *Ae. aegypti* adults (4804 female, 3203 male) were released inside and around an isolated homestead near Cairns, north Queensland, Australia. These free-range mosquitoes were free to engage in natural mosquito behavior including human blood feeding, mating, oviposition and harborage in natural micro-habitats. Females from this cohort were recaptured at known ages (2 d intervals from 1 to 29 d) and in varied physiological states, which provided an ideal sample to validate transcriptional mosquito age grading. Transcriptional profiles from the sentinel-caged mosquitoes were used to produce an age prediction model for the free-range females in an approach that may be applied to determine the age structure of wild *Ae. aegypti* populations. Mild conditions prevailed during the experiment, with average ambient temperatures of 20.9°C (range 14.7–26.5°C) in the house and 20.3°C (13.2–27.5°C) in an open sided shelter and average relative humidity of 86.2% (51.2–99.7%). Exhaustive sampling at the conclusion of the experiment collected 286 female and 11 male *Ae. aegypti*.

Dissection of free-range mosquitoes to determine physiological status showed that no female had mated by 1 d old (*n* = 10), 90% females had mated by 3 d (*n* = 10) and all females ≥5 d old were mated (*n* = 120). The first peak in blood feeding activity occurred when females were 30 hr old (±12 hr). This was reflected by high percentages of free-range females containing blood when recaptured at 3 and 5 d old (Figure S1A). The percentage of females with blood varied between 10% and 90% for subsequent samples. In some females, ovary development had advanced as far as Christophers' stage IV by 3 d old (28 hr after the first blood feeding peak; Figure S1B). The proportion of gravid females (those containing a clutch of mature, stage V ovaries) was 10% at 5 d, increased to 80% by 9 d and fell in subsequent days as females oviposited and blood feeding recommenced. For the females in which ovarian skeins could be visualized, all females ≤3 d old were nulliparous (*n* = 14) and all older females were parous (*n* = 48). The mean wing length of the free range female *Ae. aegypti* was 3.01 mm, SE = 0.01, significantly larger than previously recorded for wild *Ae. aegypti* collected under equivalent seasonal conditions in Cairns (2.85 mm, SE = 0.02; *F* = 71.40, *df* = 256, *P*<0.001).

**Figure 1:**
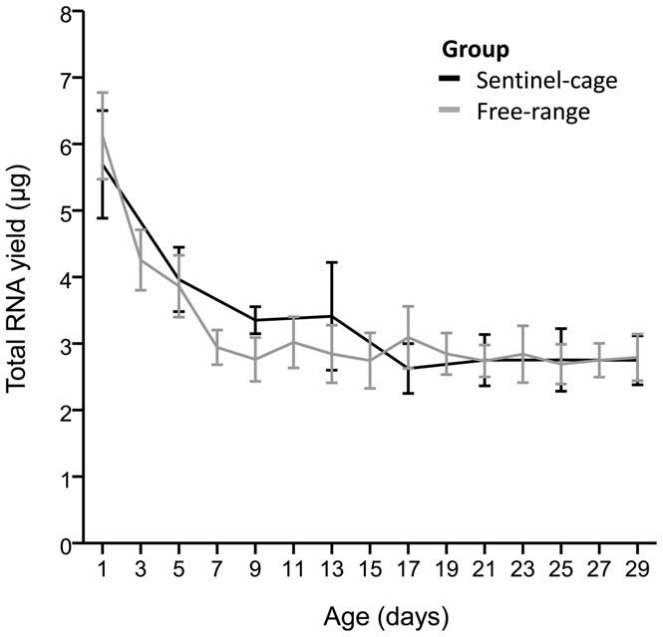
Variation with age in total RNA yield from sentinel cage and free-range *Aedes aegypti* females. Lines indicate mean (±95% CI) total RNA quantity obtained from head and thorax tissue of individual mosquitoes.

A preliminary step in the transcriptional age grading assay is the isolation of total RNA from the mosquito head and thorax and reverse transcription of a standard quantity of total RNA to cDNA. Early indication that a specimen was relatively young was gained at this stage because total RNA yield decreased with age in *Ae. aegypti* females from 1 to 5 d old, with RNA levels stabilizing in later samples (Figure 1). The total RNA yield was not significantly different between sentinel cage and free range females (ANOVA, *n* = 214, *P* = 0.13) but was strongly influenced by log-age (*P*<0.001). We then examined potential physiological factors that may influence mosquito total RNA quantity and found that as well as log-age, the yield was highly influenced by ovary development (ANOVA, *n* = 145, *P*<0.001) and wing length, measured as a proxy for body size (*P* = 0.002), but was not influenced by the presence of blood in the midgut (*P* = 0.61). The model was fitted with an interaction between blood presence and ovarian development that was not significant (*P* = 0.15). A comparison of the main effects of ovarian development showed that egg maturation was associated with an increase in total RNA quantity in the head and thorax, with the rate steadily increasing from stage I to stage IV before total RNA levels dropped at the completion of egg development at stage V (Figure S2).

Having previously identified a set of four genes that facilitated age predictions of *Ae. aegypti* (three age responsive genes; *Ae-15848*, *Ae-8505* and *Ae-4274* and a housekeeping gene, *Ae-RpS17* [14]), we applied Taqman-probes targeting transcripts of these genes to streamline the determination of mosquito age. *Ae-RpS17* and *Ae-15848* are more highly expressed than *Ae-4274* and *Ae-8505*, with cycle threshold (Ct) values differing by approximately 10 cycles. Attempts were made to primer limit the amplification of *Ae-RpS17* and *Ae-15848*, and allow for the efficient amplification of the other two amplicons in later cycles. However, the co-amplification of all amplicons could not be achieved despite efforts to optimize MgCl_2_ and primer concentration. The similarity of transcript abundance between *Ae-RpS17* and *Ae-15848*, and *Ae-4274* and *Ae-8505* allowed for the development of a duplex Taqman assay without the obstacle of preferential amplification of highly abundant cDNA templates. Duplex assays were validated across a 10^7^-fold dilution series of template abundance. Standard curves were constructed for the single and duplex Taqman assays, by plotting linear regressions of Ct value against the log concentration of linearized plasmid template. The slopes of the regression (PCR efficiency) for the single and duplex standard curves were determined to be equivalent by ANCOVA. *Ae-4274* was the only amplicon where the duplex reactions were significantly different from the single-plex reactions across the 10^7^-fold dynamic range examined (*n* = 53, *df* = 1, *P*<0.05).

Age related variation was evident from the transcriptional profiles of the age responsive genes in *Ae. aegypti* head and thorax tissue (Figure S3). Transcription is represented as the log contrast of the qPCR Ct values of the gene relative to the housekeeping gene (*Ae-RpS17*). Log contrasts are inverse measures of transcript abundance, increasing as transcription is decreasing. Log contrast values describing the transcription of *Ae-15848* showed a four-fold increase from 1 to 29 d old (Figure S3A). The effect of log-age was highly significant (ANOVA, *n* = 217, *P*<0.001) but there were no differences between sentinel cage and free-range females (*P* = 0.73). For *Ae-8505*, log contrast values increased rapidly from 1 to 3 d and increased at a more gradual rate with age in older females (Figure S3B). Similarly, the effect of log-age was highly significant (*P*<0.001) and differences between caged and free-range mosquitoes were not significant (*P* = 0.23). Log contrast values for *Ae-4274* decreased gradually with age (Figure S3C); however the effect of log-age was highly significant (*P*<0.001). No significant differences were observed between caged and free-range mosquitoes (*P* = 0.65). We then examined the influence of the measured physiological factors on the transcription of the genes as a further test of their robustness as biomarkers of age. We analyzed the effects of log-age, wing length, presence of blood in the midgut, ovary development stage and an interaction between blood presence and ovary development on the log contrast values of *Ae-15848*, *Ae-8505* and *Ae-4274*. The effect of log-age was highly significant for all genes, however; none of these factors or the interaction was significant (Table S2).

The sentinel cage females were used as training samples to establish an age-prediction calibration model. The log contrast values for each female were entered into canonical redundancy analysis to produce a single redundancy variate. The analysis indicated that 68.8% of the variance in the gene transcription measures was explained by age. An age prediction calibration model was constructed from the regression of the redundancy variate for each female against adult age (Figure S4). An important outcome was that there was a strong linear component to the model (Linear regression *R^2^* = 0.688, *n* = 72, *P*<0.001).

**Figure 2:**
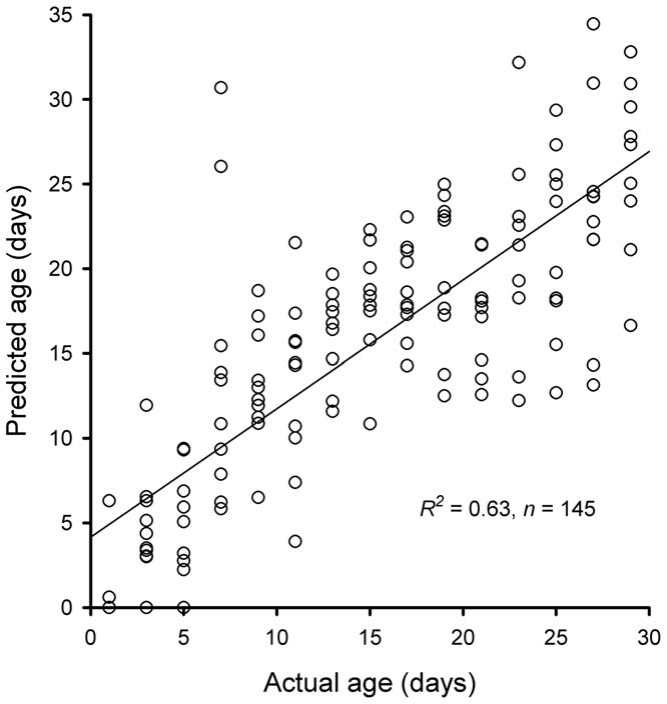
Age predictions of free-range *Aedes aegypti* from the sentinel-cage derived calibration model. Values are age predictions for individual free-range females (*n* = 145) derived from nonparametric bootstrap methods. Solid line, least squares linear regression line. Dashed line, predicted equals actual age line.

The free-range *Ae. aegypti* females were treated as age-blinded test specimens. Transcription was quantified and log contrasts were calculated as for the sentinel cage mosquitoes. Canonical redundancy analysis was used to derive a redundancy variate for each individual and age was predicted using a bootstrap procedure that applied inverse regression of the sentinel cage model from this redundancy variate. The predicted ages were then compared to the actual ages of these females, known from the time of recapture. This comparison showed a strong, near-linear relationship between the predicted ages and the actual ages of the free-range females (Figure 2; *R^2^* = 0.62, *n* = 145, *P*<0.001). Negative ages were predicted, because the normal distribution used to model age is not constrained to positive values. A Poisson model with logarithmic link function was applied to the training and test datasets because predicted values were constrained to positive values; however no overall gains in precision or accuracy were made over the redundancy variate model (Fig. S5A). Interestingly, inclusion of total RNA yield as a predictor variable increased the fit of the Poisson regression model (Fig. S5B); however no gains were made by including total RNA in the redundancy variate model (Fig. S5C). Negative predictions were therefore manually reset to zero days. Moderate accuracy of age predictions was achieved, with 31.0% of ages predicted to within 2 d of the actual age, 55.9% to within 4 d and 77.2% to within 6 d. However, 8.3% of predictions were greater than 10 d from the actual age. Two of these samples, obvious from Fig. 2, were 7 d old females that had log contrast values for *Ae-4274* that were greater than three standard deviations from the mean of all free-range females. None of the physiological factors measured (blood digestion, ovary development and body size) had a significant effect on the age prediction accuracy (Table S3). However, slight age prediction bias was observed as indicated by a significant effect of age on the error (*P*<0.001). Inspection of age prediction residuals against predicted age (Fig. S6) showed no clear trend indicating that an appropriate model had been fitted.

**Figure 3:**
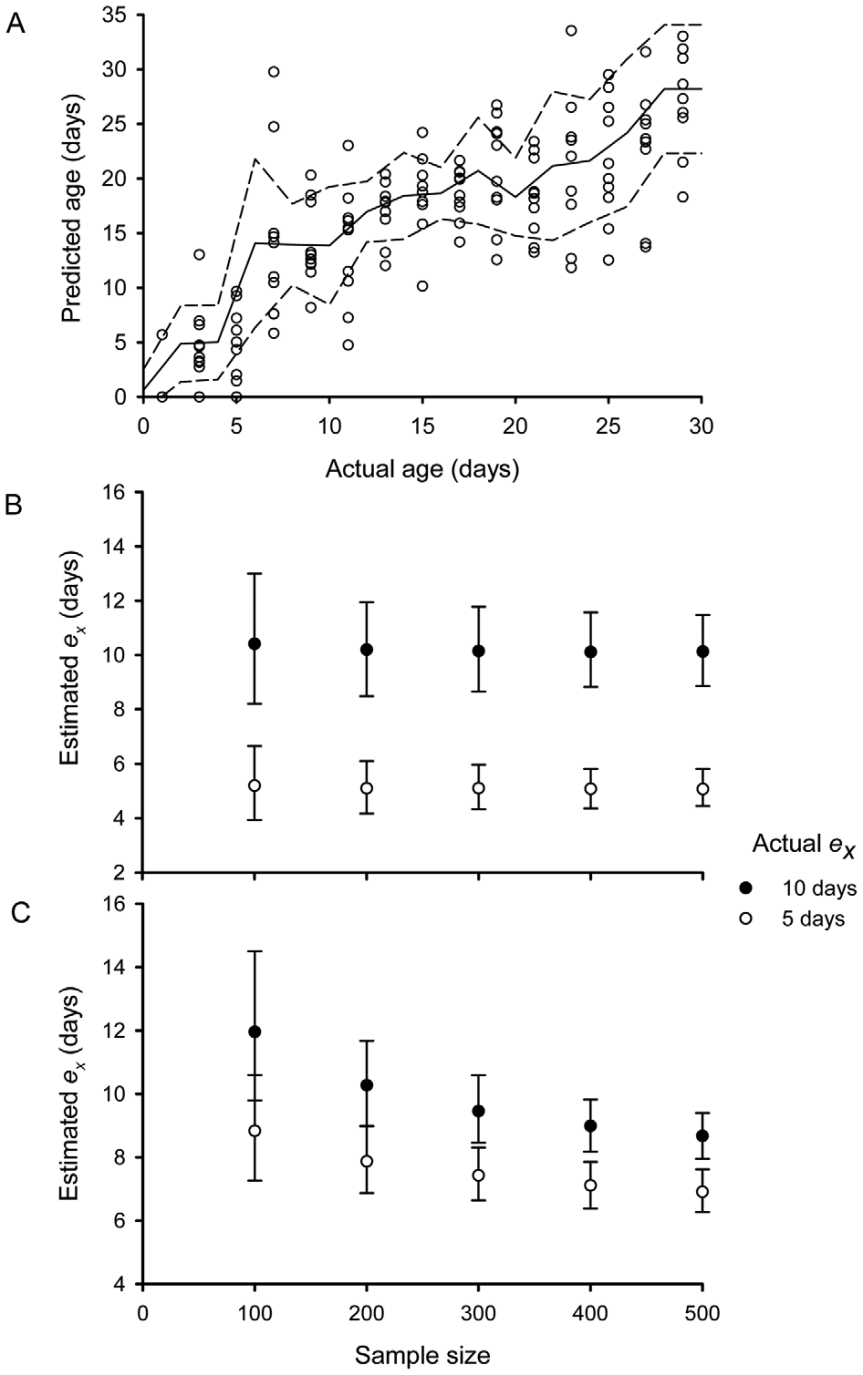
Assessments of the capacity of transcriptional age grading to differentiate two mosquito populations with differing mean life expectancies. A. Experimental age predictions showing interpolated mean (solid line) and standard deviations (broken lines) that were used to generate cumulative normal distributions for each age. Ages were then randomly selected from these distributions and assigned to each individual sampled from a population defined by a given *e_x_* value. Random samples of 100, 200, 300, 400 and 500 female mosquitoes were modeled. Symbols indicate mean *e_x_* values for 999 replicates and bars show 95% confidence limits. B. Estimated population *e_x_* values generated from random samples of different sizes when ages were predicted without error. C. Estimated population *e_x_* values from different sample sizes when mosquito ages were predicted from the experimental error distributions for age predictions achieved in this study. Symbols indicate mean *e_x_* values and bars show 95% confidence limits.

Monte Carlo simulation enabled us to model the application of transcriptional age grading to the estimation of population *e_x_* values using the experimental error distributions obtained from the release-recapture experiment (Fig. 3A). First, we determined the error distribution that would be expected through sampling error alone, assuming that all mosquitoes sampled were age graded with 100% accuracy (Fig. 3B). As expected, very little bias was observed in the estimates of *e_x_*. The estimates for the two populations were clearly differentiated and the precision of the estimates increased with increasing sample sizes. Second, we examined the estimates of *e_x_* from these same populations that would result if age estimates were derived by applying transcriptional age grading. For the population with *e_x_* of 10 d, the predicted *e_x_* values were not significantly different from the actual *e_x_* for sample sizes of 100–300 mosquitoes but an underlying bias towards underestimation of *e_x_* became evident at greater sample sizes (Fig. 3C). However, estimates for the *e_x_* = 5 days population were significantly greater than the actual *e_x_* values at all sample sizes. An important outcome was that the two populations could be significantly differentiated from each other when estimates of *e_x_* were based on the age predictions of >200 mosquitoes.

## Discussion

We have shown that age grading mosquitoes based on gene transcription can be successfully applied to adult female *Ae. aegypti* maintained in the wild. The ability to determine the age of female *Ae. aegypti* to an accuracy of ±6 d for 72.2% of females under field conditions is a valuable asset for investigations of mosquito population age structure. One of many applications of mosquito age grading is for the assessment of the efficacy of mosquito control interventions, whether testing the capacity of an entomopathogenic micro-organism to shorten the mean life expectancy of a mosquito population, or to test the fitness of a transgenic mosquito with impaired ability to transmit pathogens in comparison to wild type mosquitoes. We demonstrated the capacity for transcriptional age grading to differentiate between two populations of mosquitoes having mean life expectancies of 5 and 10 d. A 2-fold difference was chosen to test the capacity of the model to identify a 50% lifespan reduction of *Ae. aegypti* females that is expected to result from a dengue control intervention based on *Wolbachia* intracellular bacteria [6],[8],[9]. Although bias was evident from absolute estimates of *e_x_* derived from transcriptional age estimates, a relative comparison of the predicted life expectancies enabled successful differentiation of the populations.

We have increased the throughput of transcriptional age grading by applying duplex Taqman probe assays to the quantification of gene transcripts which is highly desirable for investigations of mosquito population age structure in which hundreds of specimens potentially require age grading. We applied a stringent test for validation of transcriptional age grading by releasing free-range *Ae. aegypti*, thereby allowing mosquitoes to engage in normal mosquito behavior, including blood feeding, dispersal in search of natural oviposition and resting sites and thermoregulation through harborage in typical microhabitats. As a result, females were recaptured in various physiological states at each age. However, of several physiological factors assessed by dissection (presence of blood in the midgut, ovary development and body size), none affected the transcriptional profiles of the age responsive genes or age prediction accuracy. Similarly, transcriptional profiles did not differ between females from the sentinel cages or the free-range females which is important from an applied perspective as it indicates that age prediction models for wild caught mosquitoes can be calibrated on known-age mosquitoes maintained in captivity.

There was some decrease in accuracy when compared to our previous application of the three gene age prediction model to *Ae. aegypti* maintained in field cages up until 19 days old [14]. However, we have increased the sample size of test specimens in the present study (30 to 145) and have extended the maximum age of samples to 29 d here. A Poisson log link model and alternative combinations of genes and total RNA yield predictor variables were tested; however, no improvements in accuracy were achieved over the three gene redundancy variate model. Outliers were attributed to aberrant gene transcription values, in some cases greater than three standard deviations from the mean of the group. The reasons for these extreme observations could not be determined. However, these outliers comprised less than 5% of all predictions. Environmental variation or other physiological factors not measured could account for the differences. The free-range females were larger than wild specimens collected under equivalent seasonal conditions; however, investigations in the laboratory have shown that transcription of the age responsive genes is robust to body size variation induced by varying the quantity of food provided to larvae (LEH, unpublished data).

We have also shown that a yield of total RNA from the head and thorax of an adult female above a threshold (4.4 µg in our experiments) provides early indication that the specimen is a newly emerged, <3 d old adult (a teneral adult). A 40% decrease in total RNA abundance with age from 1 to 7 d old has been previously observed from *Ae. aegypti* females [20]. High levels of total RNA in 1–2 d old adults followed a peak in total RNA abundance during the pupal stage, and were probably a residual effect of high transcription rates during metamorphosis. However, age related changes to total RNA yields have not previously been utilized for mosquito age grading assessments. In *D. melanogaster*, total RNA abundance decreased by 60% at a constant rate from 2 to 40 d of age [21]. Total ribosomal RNA, transfer RNA and mRNA levels decrease rapidly from emergence to 10 d in *Drosophila* and are thought to be due to down-regulation of RNA polymerase I, II and III mediated transcription [22]. Total RNA yield is dependent on the method of extraction used and we have shown that total RNA yield in the adult head and thorax increases with body size and during ovary development. These factors should be standardized in age grading assessments of wild mosquitoes based on total RNA yield.

The ability to accurately determine the ages of wild caught mosquitoes is crucial for investigations into the population dynamics and vulnerabilities of important mosquito vectors. In particular, the capacity to differentiate two populations of mosquitoes based on changes to mean life expectancy will be critical for evaluating new dengue control interventions targeting mosquito longevity. We estimate that adopting the Taqman probe multiplexing approach saves approximately 20% in reagent and 30% time savings when compared to the equivalent Sybr green based approach. The cost of reagents required to derive age predictions was approximately US$7.5 per mosquito. Improvements in accuracy and throughput can be expected if additional age responsive genes are identified and included in the model. Optimized Taqman reactions could enable these transcripts to be measured in triplex or quadriplex qPCR assays. The genes on which our age prediction assay is based are conserved and therefore there is a large potential for the development of transcriptional age grading methods for other insect vectors of tropical diseases.

## Supporting Information

Figure S1 Physiological condition of free-range *Aedes aegypti* females at the time of recapture. A. Presence of blood in the midgut. B. Ovary development category (Christophers' stage).
(0.61 MB TIF)

Figure S2 Effect of ovarian development on the total RNA yield from the head and thorax of *Aedes aegypti* females. Columns show means and error bars indicate SE. Bars sharing the same letter are not significantly different (*P*>0.05).
(0.06 MB TIF)

Figure S3 Log contrast variables describing transcription of age responsive genes from sentinel cage and free-range *Aedes aegypti*. A. *Ae-15848* (*Calcium binding protein*), B. *Ae-8505* (*Pupal cuticle protein 78E*) and C. *Ae-4274* (*Cell division cycle 20* [*cdc20*; *fizzy*]). Values are means of the log contrast of the gene relative to the reference gene (*Ae-RpS17*) for individual females. Bars indicate SE. An increasing trend indicates decreasing transcription.
(0.40 MB TIF)

Figure S4 Age prediction calibration model for individual *Aedes aegypti* females. The three log contrast gene expression measures were entered into canonical redundancy analysis, a procedure that reduces the dimensionality of multivariate data by calculating new variables (redundancy variates). Each redundancy variate is a linear combination of the log contrast variables that maximises the correlation with mosquito age. Points indicate the values for the first redundancy variate for individual sentinel cage females. The regression of the first redundancy variate with age (line) represents the calibration model used to predict the ages of test mosquitoes.
(0.08 MB TIF)

Figure S5 Age predictions of free-range females resulting from alternative models to the redundancy variate three-gene model. A. Poisson with log link model using three genes as input variables. B. Poisson with log link model using three genes and total RNA as input variables. C. Redundancy variate model with three genes and total RNA yield as predictor variables.
(0.52 MB TIF)

Figure S6 Age prediction residuals from predictions of the free-range females from the sentinel cage redundancy variate model.
(0.10 MB TIF)

Table S1 Primer and Taqman probe sequences used for the transcriptional age-grading assay. Taqman probes are dual-labelled with 3′ and 5′ modifications. The 3′ modifications are coloured fluorophores; FAM, CAL Orange, CAL Red and Quasar 670. The 5′ modifications (Black Hole Quenchers - BHQ) are molecules that are specifically design to minimise fluorescence of the fluorophore by frequency resonance energy transfer.
(0.03 MB DOC)

Table S2 Evaluation of the robustness of the transcription predictor variables of mosquito age against physiological variation. ANOVA was performed on the log contrast variables of free-range females with presence of blood and ovary development stage (Christophers' stage) as factors and log-age and wing length as covariates (*n* = 141).
(0.03 MB DOC)

Table S3 Evaluation of the effects of age and physiological variation on the accuracy of age predictions. ANOVA was performed on the age prediction residuals (predicted minus actual age) of recaptured females with blood presence and ovary development stage (Christophers' stage) as factors and log-age and wing length as covariates (*n* = 141).
(0.03 MB DOC)
