# Supplementary figures and images for: Field Validation of a Transcriptional Assay for the Prediction of Age of Uncaged Aedes aegypti Mosquitoes in Northern Australia
Source: PLoS Negl Trop Dis. 2010 Feb 23;4(2):e608. doi: 10.1371/journal.pntd.0000608 (PMC2826399; doi:10.1371/journal.pntd.0000608)

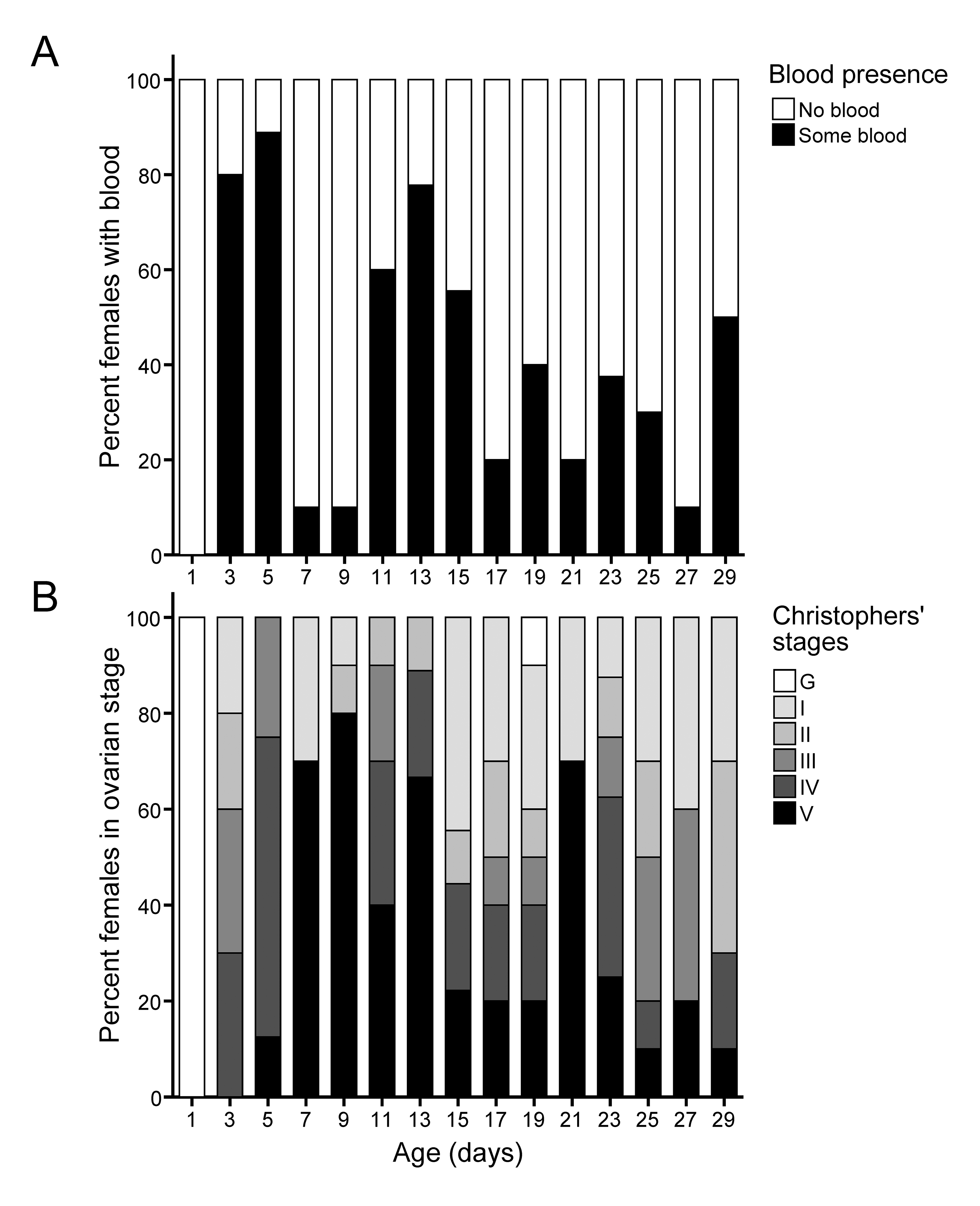

Supplement: Figure S1 — Physiological condition of free-range Aedes aegypti females at the time of recapture. A. Presence of blood in the midgut. B. Ovary development category (Christophers' stage). (0.61 MB TIF) [file pntd.0000608.s001.tif]

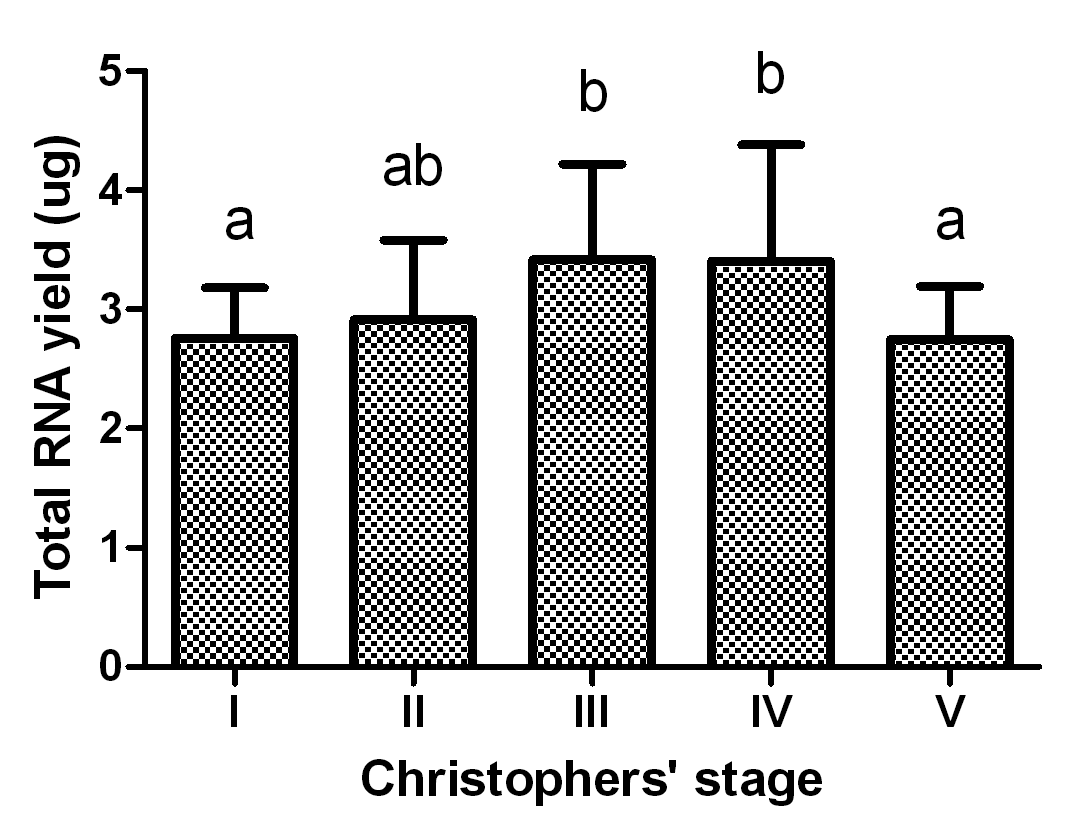

Supplement: Figure S2 — Effect of ovarian development on the total RNA yield from the head and thorax of Aedes aegypti females. Columns show means and error bars indicate SE. Bars sharing the same letter are not significantly different (P>0.05). (0.06 MB TIF) [file pntd.0000608.s002.tif]

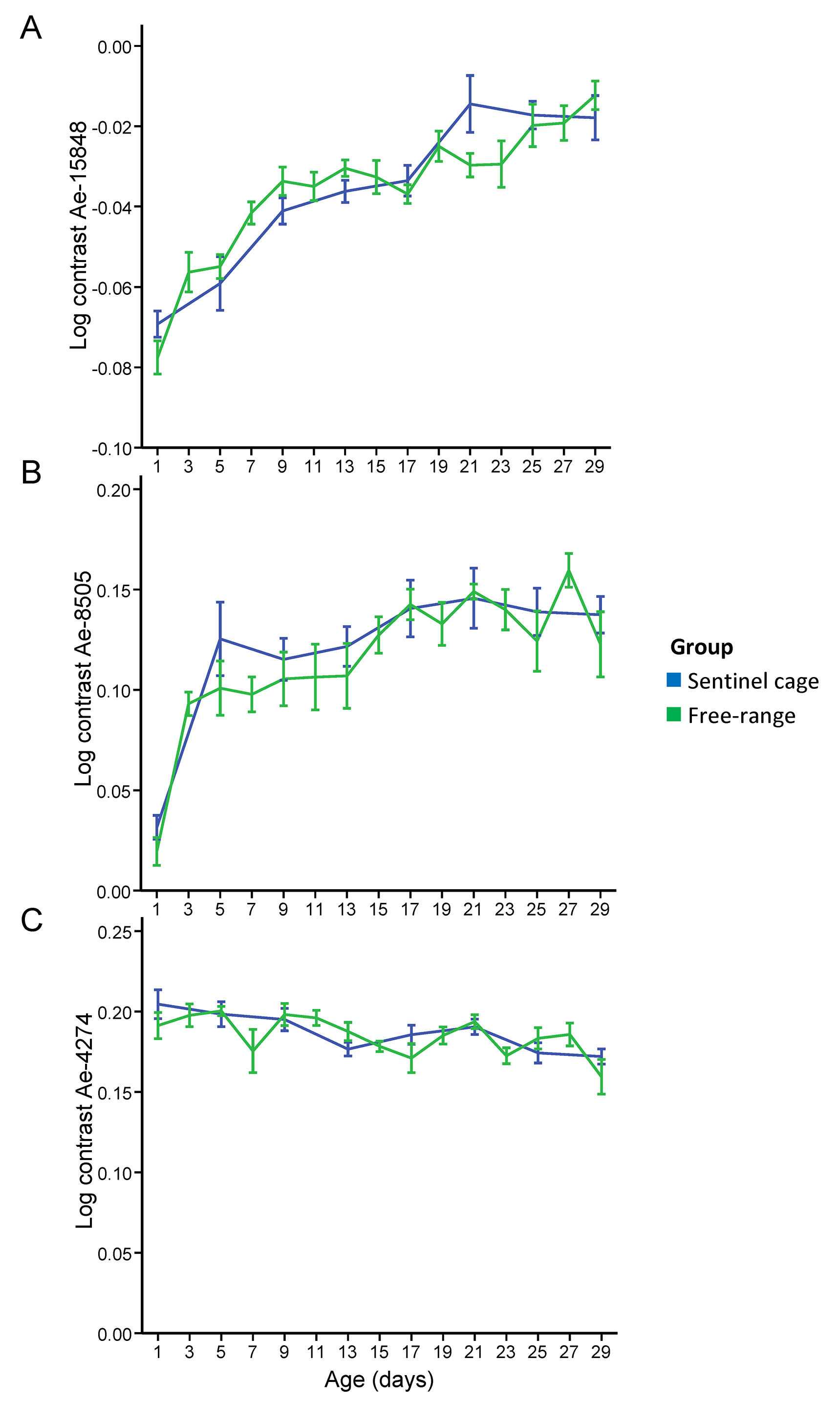

Supplement: Figure S3 — Log contrast variables describing transcription of age responsive genes from sentinel cage and free-range Aedes aegypti. A. Ae-15848 (Calcium binding protein), B. Ae-8505 (Pupal cuticle protein 78E) and C. Ae-4274 (Cell division cycle 20 [cdc20; fizzy]). Values are means of the log contrast of the gene relative to the reference gene (Ae-RpS17) for individual females. Bars indicate SE. An increasing trend indicates decreasing transcription. (0.40 MB TIF) [file pntd.0000608.s003.tif]

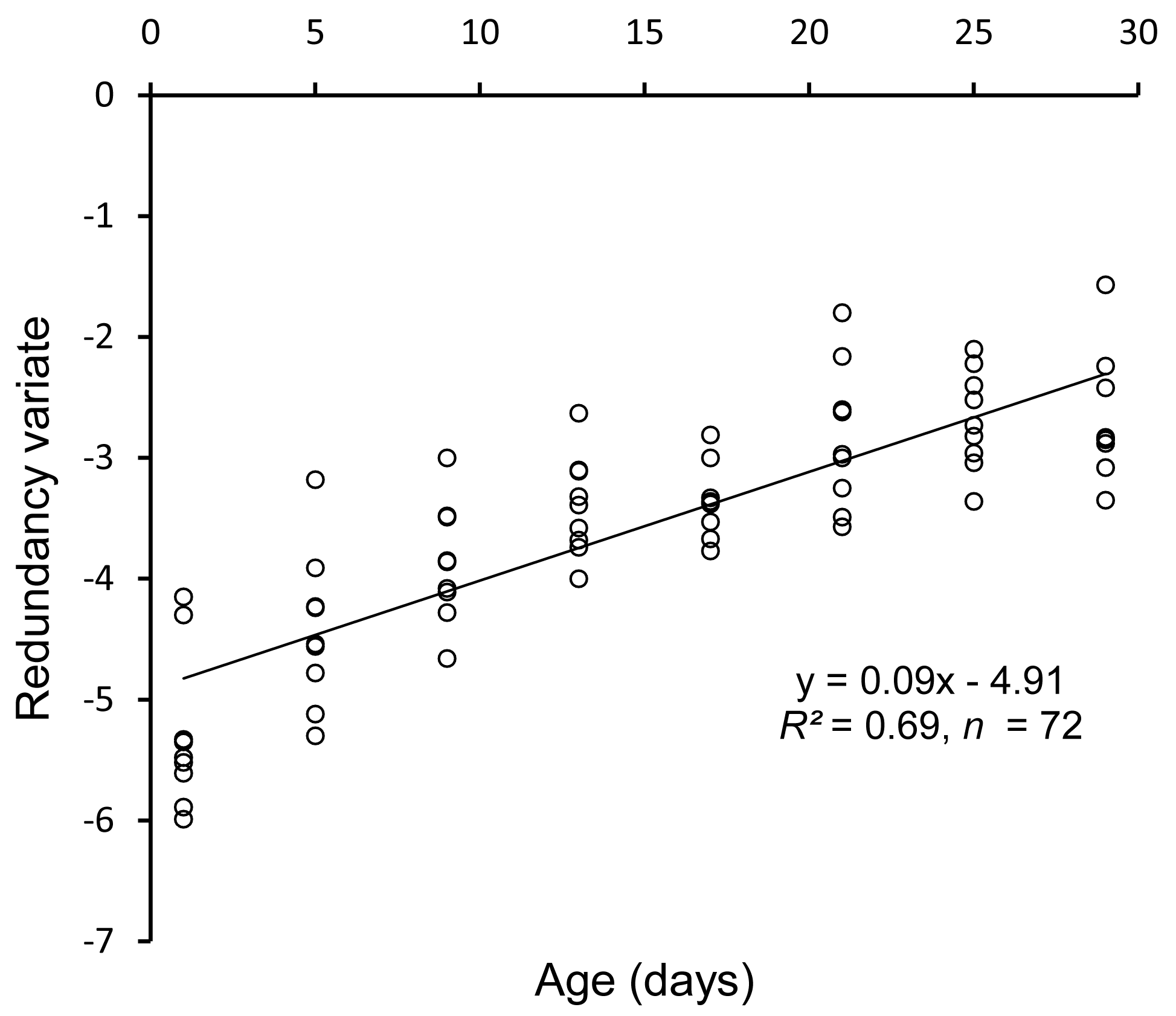

Supplement: Figure S4 — Age prediction calibration model for individual Aedes aegypti females. The three log contrast gene expression measures were entered into canonical redundancy analysis, a procedure that reduces the dimensionality of multivariate data by calculating new variables (redundancy variates). Each redundancy variate is a linear combination of the log contrast variables that maximises the correlation with mosquito age. Points indicate the values for the first redundancy variate for individual sentinel cage females. The regression of the first redundancy variate with age (line) represents the calibration model used to predict the ages of test mosquitoes. (0.08 MB TIF) [file pntd.0000608.s004.tif]

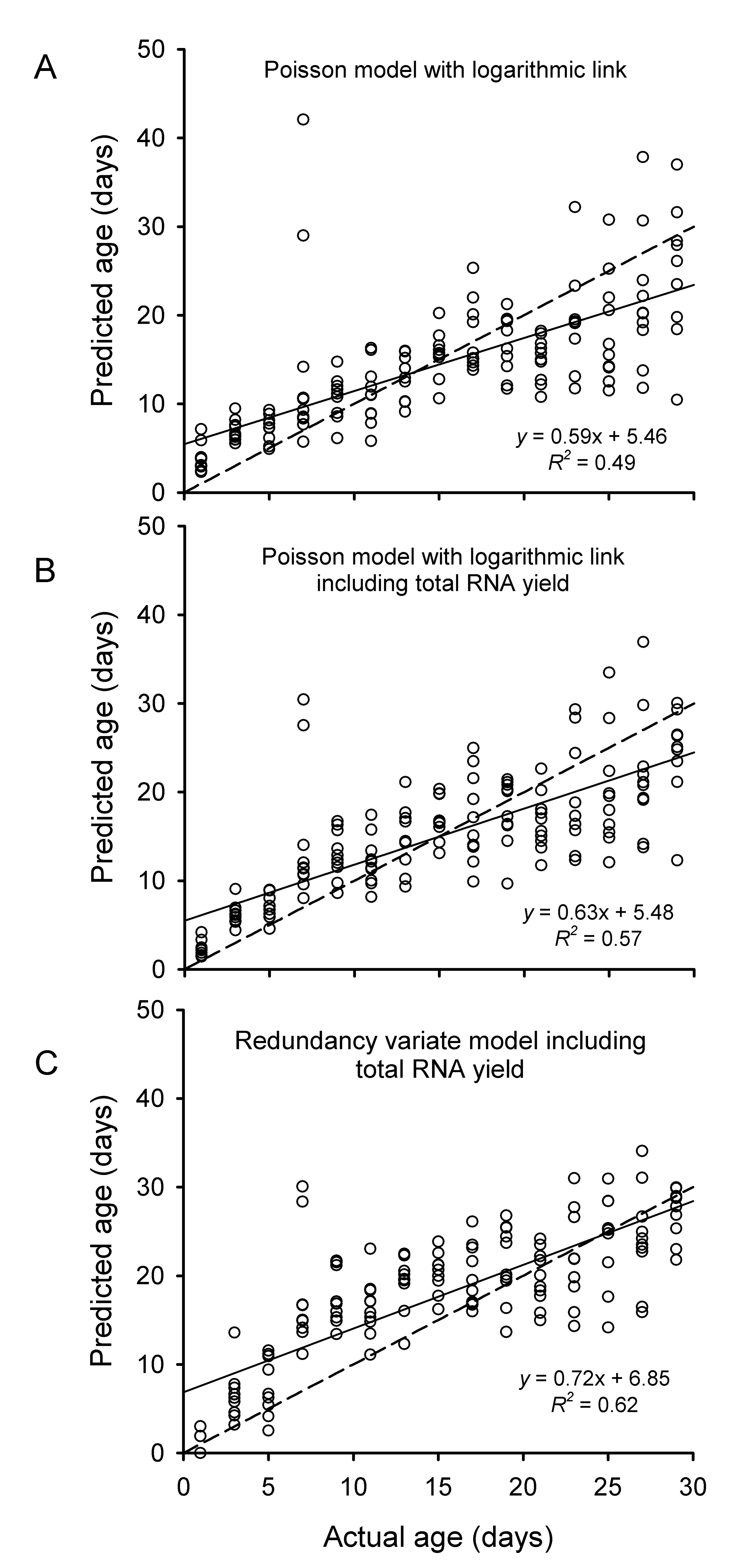

Supplement: Figure S5 — Age predictions of free-range females resulting from alternative models to the redundancy variate three-gene model. A. Poisson with log link model using three genes as input variables. B. Poisson with log link model using three genes and total RNA as input variables. C. Redundancy variate model with three genes and total RNA yield as predictor variables. (0.52 MB TIF) [file pntd.0000608.s005.tif]

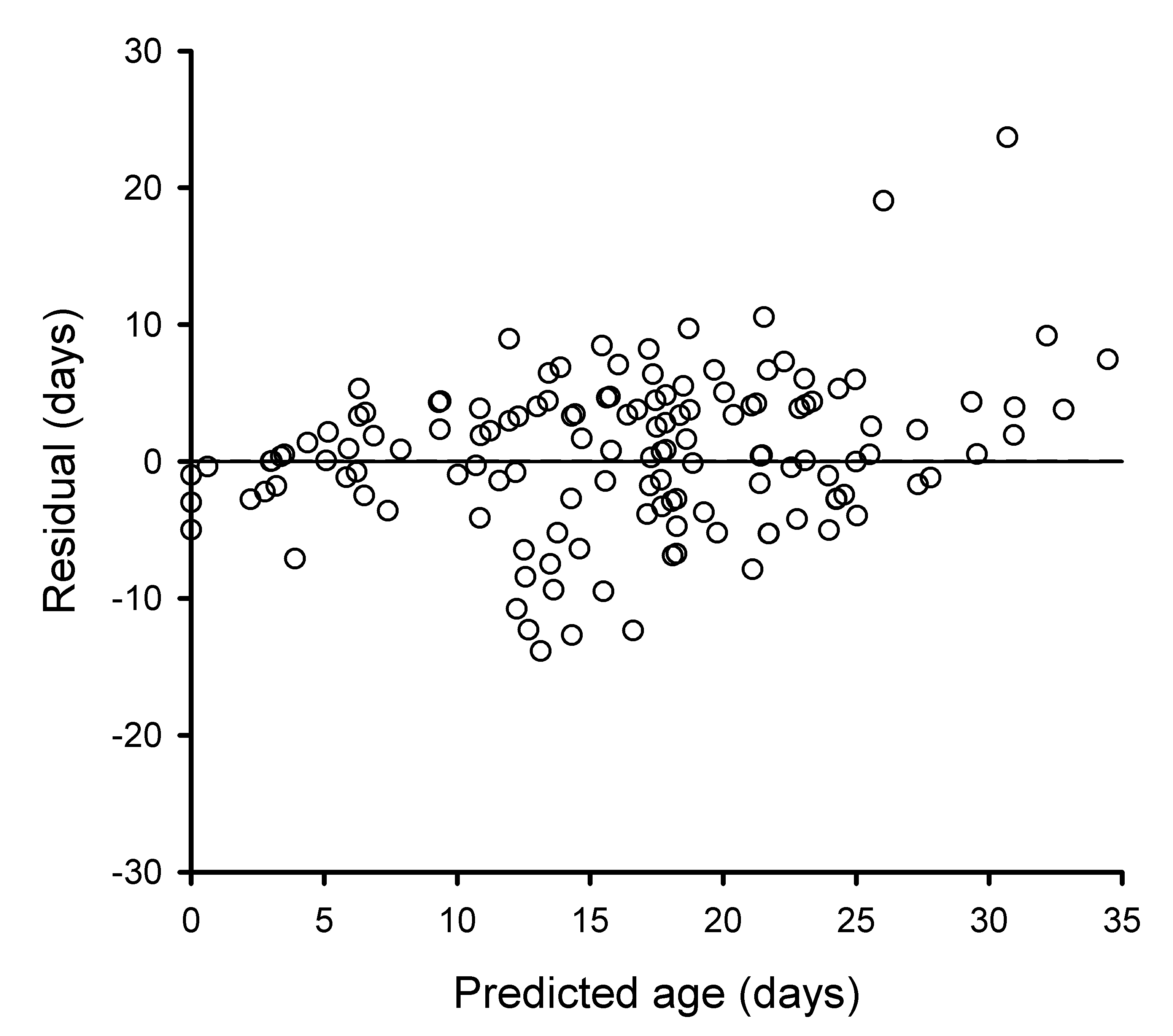

Supplement: Figure S6 — Age prediction residuals from predictions of the free-range females from the sentinel cage redundancy variate model. (0.10 MB TIF) [file pntd.0000608.s006.tif]
