# Supplementary material for: Field Validation of a Transcriptional Assay for the Prediction of Age of Uncaged Aedes aegypti Mosquitoes in Northern Australia
Source: PLoS Negl Trop Dis. 2010 Feb 23;4(2):e608. doi: 10.1371/journal.pntd.0000608 (PMC2826399; doi:10.1371/journal.pntd.0000608)
Supplement: Table S1 — Primer and Taqman probe sequences used for the transcriptional age-grading assay. Taqman probes are dual-labelled with 3′ and 5′ modifications. The 3′ modifications are coloured fluorophores; FAM, CAL Orange, CAL Red and Quasar 670. The 5′ modifications (Black Hole Quenchers - BHQ) are molecules that are specifically design to minimise fluorescence of the fluorophore by frequency resonance energy transfer. (0.03 MB DOC) [file pntd.0000608.s007.doc]

| **Gene** | **Primer/probe** | **Sequence (5' - 3')** | **Amplicon size (bp)** |
| --- | --- | --- | --- |
| *­Ae-RpS17* | RG-AeRpS17F | GGTTTCGTGACACATCT | 67 |
|  | RG-AeRpS17R | GCAGCTTGATGGAGATAC |  |
|  | RG-AeRpS17-Taqman | FAM-ATGAAGCGCCTGCGCCACTC-BHQ1 |  |
| *Ae-15848* | RG-Ae15848F | TGCTGGGTTGATGGTG | 83 |
|  | RG-Ae15848R | ATGGCTTCCTGGATAACAAC |  |
|  | RG-Ae15848-Taqman | CAL Red-CTTGCCCTCGACGACGGTGG-BHQ2 |  |
| *Ae-8505* | RG-Ae8505F | GGGTGGATTCCAACCA | 60 |
|  | RG-Ae8505R | ATTTGCGGCGGGATAG |  |
|  | RG-Ae8505-Taqman | Quasar 670-CAGGGAGACCATCTGCCAACTCC-BHQ2 |  |
| *Ae-4274* | RG-Ae4274F | GTTTGCGGTCTGAAGTG | 81 |
|  | RG-Ae4274R | CGTGCTCCATACATTGAC |  |
|  | RG-Ae4274-Taqman | CAL Orange-TCCTCGGATGGGAAACATCTTGC-BHQ1 |  |
