# Supplementary material for: Field Validation of a Transcriptional Assay for the Prediction of Age of Uncaged Aedes aegypti Mosquitoes in Northern Australia
Source: PLoS Negl Trop Dis. 2010 Feb 23;4(2):e608. doi: 10.1371/journal.pntd.0000608 (PMC2826399; doi:10.1371/journal.pntd.0000608)
Supplement: Table S2 — Evaluation of the robustness of the transcription predictor variables of mosquito age against physiological variation. ANOVA was performed on the log contrast variables of free-range females with presence of blood and ovary development stage (Christophers' stage) as factors and log-age and wing length as covariates (n = 141). (0.03 MB DOC) [file pntd.0000608.s008.doc]

| **Factor** | ***df*** |  | **Gene** |  |  |  |  |  |  |  |
| --- | --- | --- | --- | --- | --- | --- | --- | --- | --- | --- |
|  |  |  | ***Ae-154848*** |  |  | ***Ae-8505*** |  |  | ***Ae-4274*** |  |
|  |  |  | ***F*** | ***P*** |  | ***F*** | ***P*** |  | ***F*** | ***P*** |
| **Blood presence** | 1 |  | 1.90 | 0.17 |  | 0.02 | 0.90 |  | 0.07 | 0.79 |
| **Ovarian development** | 4 |  | 1.57 | 0.19 |  | 1.25 | 0.29 |  | 0.74 | 0.57 |
| **Log-age** | 1 |  | 102.97 | < 0.001 |  | 20.33 | <0.001 |  | 8.69 | 0.004 |
| **Body size (wing length)** | 1 |  | 0.96 | 0.33 |  | 0.32 | 0.57 |  | 0.98 | 0.33 |
| **Blood * ov. development** | 4 |  | 2.23 | 0.07 |  | 1.07 | 0.38 |  | 0.47 | 0.76 |
