# Supplementary material for: Field Validation of a Transcriptional Assay for the Prediction of Age of Uncaged Aedes aegypti Mosquitoes in Northern Australia
Source: PLoS Negl Trop Dis. 2010 Feb 23;4(2):e608. doi: 10.1371/journal.pntd.0000608 (PMC2826399; doi:10.1371/journal.pntd.0000608)
Supplement: Table S3 — Evaluation of the effects of age and physiological variation on the accuracy of age predictions. ANOVA was performed on the age prediction residuals (predicted minus actual age) of recaptured females with blood presence and ovary development stage (Christophers' stage) as factors and log-age and wing length as covariates (n = 141). (0.03 MB DOC) [file pntd.0000608.s009.doc]

| **Factor** | ***df*** | ***F*** | ***P*** |
| --- | --- | --- | --- |
| Blood presence | 1 | 0.10 | 0.75 |
| Ovarian development | 4 | 0.62 | 0.65 |
| Log-age | 1 | 12.88 | < 0.001 |
| Body size (wing length) | 1 | 2.14 | 0.15 |
| Blood * ov. development | 4 | 2.08 | 0.09 |
